# Supplementary material for: A Novel Nanoporous Adsorbent for Pesticides Obtained from Biogenic Calcium Carbonate Derived from Waste Crab Shells
Source: Nanomaterials (Basel). 2023 Nov 28;13(23):3042. doi: 10.3390/nano13233042 (PMC10707880; doi:10.3390/nano13233042)
Supplement: Supplementary file 1 [file nanomaterials-13-03042-s001.zip › nanomaterials-2727345-supplementary.pdf]

nanomaterials

Article

A novel nanoporous adsorbent for pesticides obtained from biogenic calcium carbonate, derived from waste crab shells

Fran Nekvapil [1,2], Adina Stegarescu [2], Ildiko Lung [2], Razvan Hirian [1], Dragoș Cosma [2], Erika Levei [3], Maria-Loredana Soran [1,2,*]

[1] Ioan Ursu Institute, Babeș-Bolyai University, 1 Kogălniceanu, 400084 Cluj-Napoca, Romania; fran.nekvapil@ubbcluj.ro (F.N.); razvan.hirian@ubbcluj.ro (R.H.)
[2] National Institute for Research and Development of Isotopic and Molecular Technologies, 67-103 Donat, 400293 Cluj-Napoca, Romania; adina.stegarescu@itim-cj.ro (A.S.); ildiko.lung@itim-cj.ro (I.L.); loredana.soran@itim-cj.ro (M-L. S.)
[3] INCDO-INOE 2000, Research Institute for Analytical Instrumentation, 67 Donat, 400296 Cluj-Napoca, Romania; Erika.levei@icia.ro (E.L.)
* Correspondence: Loredana.soran@itim-cj.ro; Tel.: (optional; include country code; if there are multiple corresponding authors, add author initials)

Supplementary material

Contents:

Figure S1
Figure S2

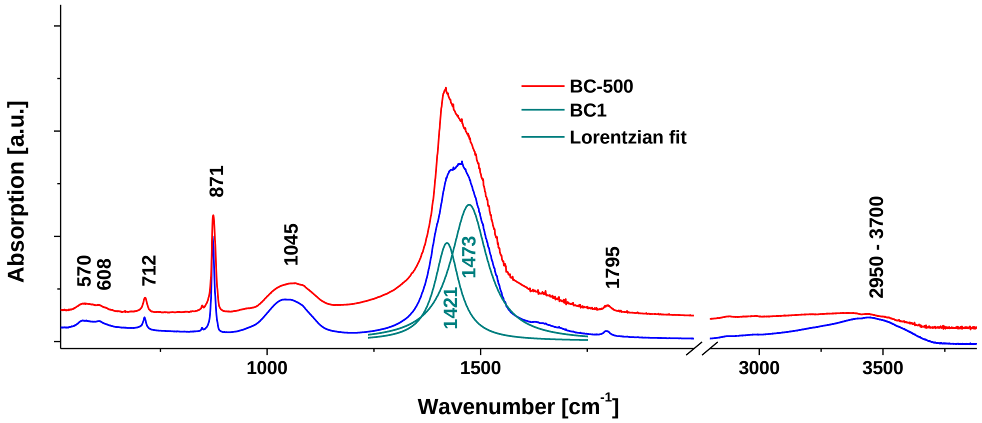

Figure S1. Comparative display of the FTIR spectra of adsorbent BC1 (heated in argon flow at 700 °C) and a batch of the same powder stock treated in air at 800 °C. The purpose of this figure is to highlight the signal of CaO and CaOH phases in air treated material, via the additional band at 3640 cm^{-1} arising from calcium hydroxide O-H stretching mode, and the red shift of the υ_3 to a 1423 cm^{-1} band relative to calcite-amorphous $CaCO_3$ mixture of BC1 which exhibits band envelope maximum at 1451 cm^{-1} characteristic to calcium oxide.

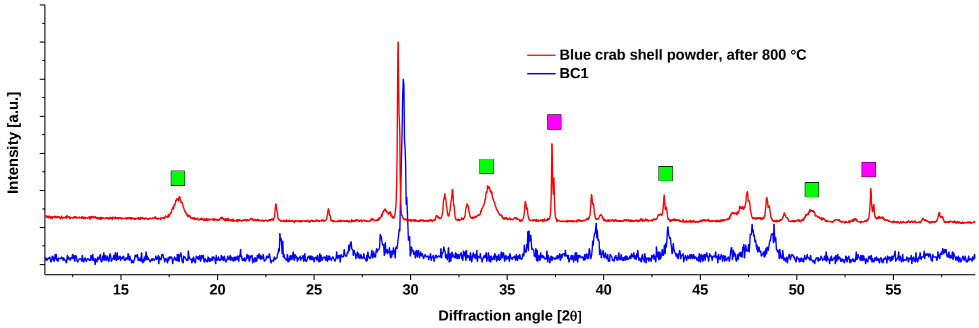

Figure S2. Comparative display of the XRD patterns of adsorbent BC1 (heated in argon flow at 700 °C) and a batch of the same powder stock treated in air at 800 °C. The purpose of this figure is to highlight the reflection peaks of CaO and CaOH phases in air treated material: CaO peaks are labelled with magenta squares, CaOH peaks are labelled with green squares.
